# A Study on Distributed Multi-Sensor Fusion for Nonlinear Systems Under Non-Overlapping Fields of View

**DOI:** 10.3390/s25134241

**Published:** 2025-07-07

**Authors:** Liu Wang, Yang Zhou, Wenjia Li, Lijuan Shi, Jian Zhao, Haiyan Wang

**Affiliations:** 1 College of Computer Science and Technology, Changchun University, Changchun 130022, Chinawanghy80@ccu.edu.cn (H.W.); 2 The Key Laboratory of Intelligent Rehabilitation and Barrier-Free for the Disabled, Changchun University, Ministry of Education, Changchun 130022, China; shilj@ccu.edu.cn; 3 Jilin Provincial Key Laboratory of Human Health Status Identification and Function Enhancement, Changchun 130022, China; 4 College of Electronic and Information Engineering, Changchun University, Changchun 130022, China

**Keywords:** different fields of view, GM-JMNS-CPHD, stochastic outlier selection (SOS), technique for order preference by similarity to ideal solution (TOPSIS)

## Abstract

To explore how varying viewpoints influence the accuracy of distributed fusion in asynchronous, nonlinear visual-field systems, this study investigates fusion strategies for multi-target tracking. The primary focus is on how different sensor perspectives affect the fusion of nonlinear moving-target data and the spatial segmentation of such targets. We propose a differential-view nonlinear multi-target tracking approach that integrates the Gaussian mixture, jump Markov nonlinear system, and the cardinalized probability hypothesis density (GM-JMNS-CPHD). The method begins by partitioning the observation space based on the boundaries of distinct viewpoints. Next, it applies a combined technique—the TOPSIS (Technique for Order Preference by Similarity to Ideal Solution) and SOS (stochastic outlier selection)—to identify outliers near these boundaries. To achieve accurate detection, the posterior intensity is split into several sub-intensities, followed by reconstructing the multi-Bernoulli cardinality distribution to model the target population in each subregion. The algorithm’s computational complexity remains on par with the standard GM-JMNS-CPHD filter. Simulation results confirm the proposed method’s robustness and accuracy, demonstrating a lower error rate compared to other benchmark algorithms.

## 1. Introduction

Sensor networks collect observation data from multiple moving targets and perspectives through their spatially distributed sensor nodes, and are widely used in drone swarm networks, multi-target tracking, and multiple research fields [1,2,3]. Determining how to integrate multi-source observation data and ensure the consistency of the target information represented by the information to be fused are the primary issues that sensor networks need to face in various fields. In fact, due to the different placement, limited detection distance, and limited detection angle, multiple sensors can have non-identical fields of view. Directly using distributed fusion algorithms under different field-of-view conditions can result in underestimating the number of targets, repeatedly calculating target motion trajectories, and a series of other problems. Therefore, exploring the problem of multi-target tracking with non-identical fields of view is crucial. Moreover, in reality, most multi-target movements are nonlinear, and nonlinear systems can more accurately describe the system’s motion state. Some filtering methods directly affect the multi-target tracking system of nonlinear targets, and the fusion effect is even worse under the influence of different perspectives. In the field of multi-target tracking, modeling and filtering for nonlinear motion targets has long been a critical research focus. Dagan [4] proposed a decentralized Bayesian fusion method for heterogeneous nonlinear systems, extending the application of the homogeneous covariance intersection algorithm. Vo [5] introduced the JM-CPHD (jump Markov–cardinalized probability hypothesis density) filter, extending the GM-CPHD (Gaussian mixture–cardinalized probability hypothesis density) framework via a double integration over both target state and motion modes. Bao [6] developed the EnSF (Ensemble Fractional Filter), capable of solving high-dimensional nonlinear filtering problems with high accuracy. Liu [7] proposed an event-triggered distributed robust cubature Kalman filter, which not only considers the heterogeneity in local estimation accuracy but also avoids computationally expensive iterations. jump Markov system models have also been employed in nonlinear tracking scenarios [8,9,10,11,12,13,14,15,16], yet these studies predominantly focus on single-sensor or single-view settings, lacking the exploration of collaborative tracking under non-overlapping fields of view (FoVs) among multiple sensors.

In the domain of distributed fusion for nonlinear multi-target tracking, several approaches have been proposed to enhance estimation performance. Wang [17] designed a distributed information filter based on maximum correntropy, leveraging consensus averaging and statistical linearization for nonlinear systems. Zhou [18] proposed a novel Poisson multi-Bernoulli mixture (PMBM) filter based on graph theory, suitable for resolving group targets with nonlinear dynamics. Lan [19] investigated zonotopic distributed fusion for a class of two-dimensional nonlinear systems in sensor networks. Hu [20] developed a distributed resilient fusion (DRF) filter for multi-sensor nonlinear singular systems with colored measurement noise. Zhao [21] addressed the challenge of distributed filtering for nonlinear multi-sensor networked systems (MSNNSs) affected by multiplicative noise, randomly varying parameters, and missing measurements. Luo [22] proposed a dynamic event-triggered fusion filter based on a sequential inverse covariance intersection strategy.

However, most of these methods rely on assumptions of consistent or partially overlapping FoVs, limiting their adaptability in scenarios with significant viewpoint disparities. Their effectiveness further deteriorates when dealing with nonlinear system models and heterogeneous sensors. Jin [23], Chen [24], and Li [25] have initiated relevant studies on distributed fusion under non-overlapping FoVs. In prior work, a Structured Fusion Model based on SOS (SFM-SOS) was introduced, which partitions the distributed fusion process into segmentation, fusion, and merging phases. After segmentation, the method computes dissimilarity matrices, affinity matrices, joint probability matrices, and output matrices with outlier probabilities to improve outlier detection accuracy under divergent views. The posterior intensity function is decomposed into multiple sub-intensities, and the target cardinality distribution in each region is reconstructed using multi-Bernoulli models [26,27]. Nonetheless, this method demonstrates only limited effectiveness in nonlinear systems and relies on manually set thresholds in the SOS algorithm, potentially introducing human-induced bias. To address these limitations, this study focuses on distributed fusion for nonlinear systems under non-overlapping FoVs and proposes an improved algorithm—Threshold-Suppressed Gaussian mixture–jump Markov Nonlinear System–CPHD (T-S-GM-JMNS-CPHD). The proposed method aims to enhance the accuracy and robustness in nonlinear, multi-view environments.

The major contributions of this chapter are summarized as follows:

1. The design of a GM-JMNS-CPHD distributed fusion framework for non-overlapping FoV scenarios: A nonlinear system modeling approach is developed from both the state-space partitioning and algorithmic perspectives to address challenges in distributed multi-sensor tracking under non-overlapping fields of view (FoVs).
2. The integration of an adaptive thresholding strategy in the SOS outlier handling module: A stochastic outlier selection (SOS) algorithm approximating the ideal solution is introduced to replace heuristic or manual threshold tuning, enhancing the robustness and adaptability of the filter in cluttered or uncertain environments.
3. Robust target cardinality estimation through intensity function decomposition and multi-Bernoulli reconstruction: The posterior intensity is partitioned into regional sub-intensities, each associated with a subspace. Cardinality distributions are estimated within each region using multi-Bernoulli modeling, thereby improving the accuracy in scenarios with varying target densities and spatial distribution.

## 2. Research Background

### 2.1. Analysis of the Impact of Non-Overlapping Fields of View on Nonlinear Moving-Target Tracking

In real-world scenarios, multiple targets often exhibit nonlinear motion patterns. However, recent research has rarely addressed the challenges posed by non-overlapping fields of view (FoVs) in multi-sensor configurations, especially in the context of nonlinear systems. Studies specifically focusing on nonlinear distributed fusion under non-overlapping FoVs remain limited.

Reference [26] discusses the impact of non-overlapping fields of view (FoVs) on (1) the results of multi-sensor fusion for nonlinear moving-target detection and (2) the state-space partitioning in nonlinear systems under distributed sensing conditions. Due to differences in sensor types, orientations, and spatial locations, each sensor may exhibit a unique field of view, which significantly affects its ability to observe nonlinear target trajectories. To address this, it is necessary to partition the target state space based on sensor-specific viewing constraints.

### 2.2. Impact of Non-Overlapping Fields of View on Distributed Fusion Results for Nonlinear Moving Targets

**Figure 1:**
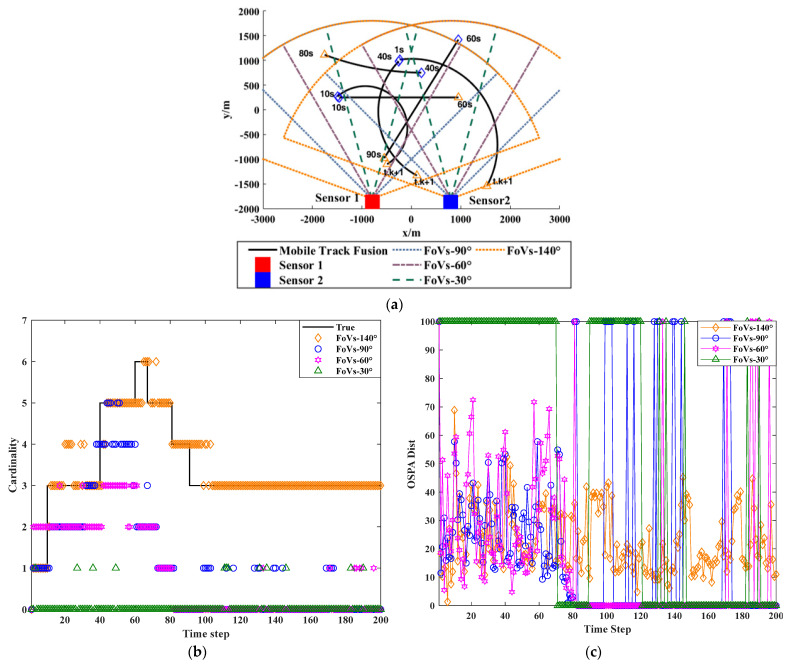
Impact of FoV on fusion results of multi-sensor tracking of nonlinear moving targets. (**a**) real track of moving target. (**b**) Cardinality estimation result of moving track. (**c**) OSPA estimation result of moving track.

In a distributed sensor network comprising two nodes, each sensor independently performs CPHD filtering for targets following distinct motion trajectories. The detection region is denoted as [−3000,3000]m×[−2000,2000]m, with each sensor having a detection radius of r=3600m, and being positioned at locations $p_{1}=[-800,-1800]$ and $p_{2}=[800,-1800]$. Both sensors are configured with a default field-of-view (FoV) center angle of 90°. Under the AA fusion scheme, the sensors’ FoVs are further adjusted to 140°, 90°, 60°, and 30° to assess the impact of the FoV variation. Performance comparisons are conducted using two key metrics: the base estimation of motion trajectories and optimal sub-pattern assignment (OSPA) error. The target trajectories are initialized according to the nonlinear Gaussian measurement model described in Table 1, where the number of time steps is truth. K = 200, and the angular velocity parameter is defined as wturn = 2π/180. The influence of varying FoV configurations on the accuracy of multi-sensor fusion is illustrated in Figure 1.

Table 1 shows the motion path, generation time, and death time of the trajectory. From the results in Figure 1 above, it can be seen that the trajectories of moving targets collected by sensors from different perspectives vary greatly, directly affecting the collection effect of nonlinear moving targets.

## 3. Distributed Fusion Algorithm Combining T-S-GM-JMNS-CPHD

### 3.1. GM-JMNS-CPHD Filter

In the work of Vo et al. [28] and Mahler et al. [29], the jump Markov–cardinalized probability hypothesis density (JM-CPHD) filter was introduced to address challenges in tracking maneuvering targets. The Gaussian mixture–CPHD (GM-CPHD) filter extends the conventional CPHD framework by augmenting single-state integration to a joint integration over both motion modes and target states, denoted here as $\ddot{x}=(x,o)$.

When the jump Markov Nonlinear System (JMNS) model is incorporated into the CPHD framework, the system omits an inherent target birth model. Therefore, both the target birth and clutter models must explicitly account for the probability distributions of newly appearing targets and false alarms. These probabilistic distributions—$p_{k+1\left|k\right.}^{B}(n)$, $p_{k+1}^{\kappa }(m)$, etc.—must satisfy the following conditions:

(1) $$\sum_{m\geq 0}n\cdot p_{k+1}^{B}(n)=\sum_{o}\int b_{k+1\left|k\right.(x,o)}dx$$

(2) $$\sum_{m\geq 0}m\cdot p_{k+1}^{\kappa }(n)=\lambda_{k+1}$$

For a positive integer *n*, the probability distribution is p(n); if *n* is negative, it is p(n)=0. For the combination coefficient $C_{n,i}$, if i>n, then $C_{n,i}=0$. In this case, x can be replaced by $\ddot{x}$.

As outlined in Reference [30], a novel implementation of the jump Markov–CPHD filter—termed the Gaussian mixture particle (GMP) method—was developed to effectively handle complex nonlinear and non-Gaussian models, especially in the context of maneuvering targets. In this framework, particle-based techniques are employed for the propagation and update of phases of local filtering, while Gaussian mixture models facilitate inter-sensor communication and information fusion. Although the literature provides a comprehensive description of the GM-JMNS-CPHD filter’s prediction and update mechanisms, the present study emphasizes the fusion strategy employed within this framework.

### 3.2. SFM-TOPSIS-SOS Fusion

Multi-view multi-target tracking primarily involves extending the local variation observed by individual sensors into a global fusion framework that captures overall discrepancies. Therefore, as a fundamental step, the fusion process across multiple sensor nodes must be addressed. Following the application of the GM-JMNS-CPHD algorithm, it is assumed that each sensor node’s local intensity function can be represented as follows:

(3) $${{\hat{D}}^{i}}_{\left(x\right)}=\sum_{i=1}^{J^{i}}\alpha_{p}^{i}N(x;m_{p}^{i},P_{p}^{i})$$

Here, *i* denotes the sensor index, while $\alpha_{p}^{i}\in (0,1)$ and N(x;m,P) represent Gaussian probability density functions (PDFs) characterized by their respective means, m, and covariances, p. Within the differential perspective, Gaussian components (GCs) from different clusters $C_{g},g\in \left\{1,2,\cdots ,G\right\}$ may either be assigned to the same cluster when viewed from a single sensor’s perspective, or simultaneously appear in distinct clusters *i* and *j* across sensors.

When GCs are considered to originate from the same cluster under a shared sensor viewpoint, the following condition holds:

(4) $${\bar{D}}_{g}^{i,j}(x,o)={\hat{D}}_{g}^{i,j}(x,o)$$

The GM-JMNS-CPHD method used in this study can be used to estimate the number of targets based on the maximum a posteriori probability (MAP) criterion, and the gth cluster obtained corresponds to the sub-content of ${\hat{D}}_{g}^{i,j}(x)$, which can be obtained from the base distribution based on the Bernoulli case approximation as follows:

(5) $${\bar{p}}_{g}^{i,j}(n)={\hat{p}}_{g}^{i,j}(n)=\prod_{p=1}^{M^{i,j}}\left(1-\alpha_{g,p}^{i,j}\right)\sigma_{M^{i,j},n}(\frac{\alpha_{g,1}^{i,j}}{1-\alpha_{g,1}^{i,j}},\cdots ,\frac{\alpha_{g,M^{i,j}}^{i,j}}{1-\alpha_{g,M^{i,j}}^{i,j}})$$

When Gaussian components (GCs) are simultaneously assigned to clusters i and j, a fusion strategy is required to reconcile the differing associations. In scenarios where GCs are distributed across distinct clusters, this study adopts a three-phase approach: splitting, fusion, and merging (SFM). This SFM strategy enables effective integration by first partitioning inconsistent components, then performing data fusion within matched regions, and finally consolidating the results to form coherent cluster representations.

#### 3.2.1. Splitting

1. Boundary segmentation of different perspectives

**Figure 2:**
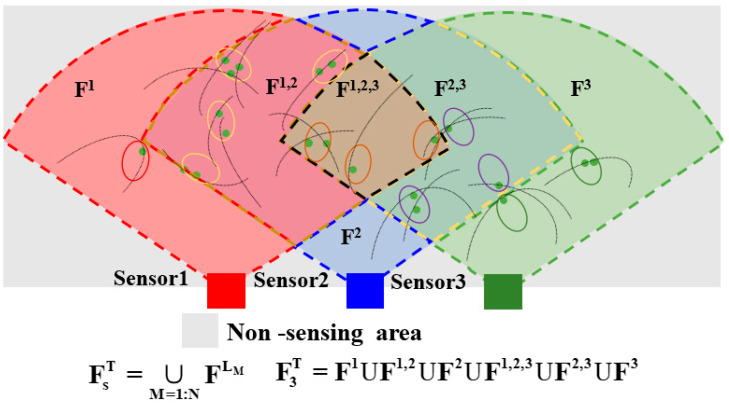
Differential viewpoint scene segmentation among different numbers of sensors.

When each sensor runs the GM-JMNS-CPHD filter over T iterations, the base distributions captured by sensors with differing viewpoints are first gathered and segmented. In this study, sensors with distinct perspectives are partitioned based on the multi-objective probability distribution (MPD). The CPHD’s ${\hat{D}}_{g}^{i,j}(x)$ region is divided into disjoint subregions according to the boundaries defined by these perspectives. The Sum of Squares (SOS) method is employed for the clustering analysis of Gaussian components (GCs), and SOS-driven anomaly detection ensures that GCs from different sensor nodes are correctly grouped into the same cluster. This accurate clustering allows the base distribution to faithfully represent the target number distribution within each corresponding region, while preserving the integrity of the original base distribution. This approach effectively mitigates fusion inaccuracies caused by underestimation—due to target points being discarded after fusion—and overestimation—caused by target points being duplicated within the sensor fusion density. Figure 2 illustrates the division of the scene into different sensor groups according to their varying viewpoints.

2. The Technique for Order Preference by Similarity to Ideal Solution–Stochastic Outlier Selection (TOPSIS-SOS)

**Figure 3:**
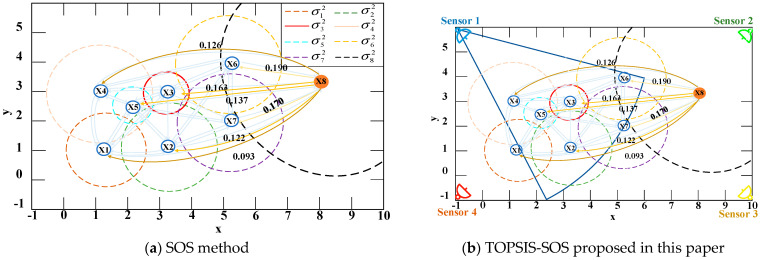
Decision-making for different clustering methods.

To determine whether Gaussian components (GCs) from different sensor nodes should be grouped into the same cluster, a cluster analysis of GCs is conducted. Figure 3a illustrates the SOS algorithm applied for the GC cluster analysis. However, SOS relies on manually set thresholds, which are often based on subjective judgment or experience, introducing uncertainty and potential bias. Moreover, in cases where data exhibit complex associations or nonlinear relationships, manually defined thresholds and distance constraints fail to capture these intricacies, thereby impacting the accuracy of the final clustering decisions.

To address these limitations, this paper abandons manual threshold setting and introduces an ordering method combining the TOPSIS with SOS, as depicted in Figure 3b. This approach utilizes the probability values and the number of sensors covering each target as the primary input data.

Taking sensor 1 as an example, it detects seven targets (X1 through X7), with boundary points identified as X1, X6, and X7. The compiled outlier probabilities and sensor coverage counts are summarized in Table 1. Here, the outlier probability is calculated using Equation (6). Additionally, the notation “+0.5” appended to the sensor coverage count indicates that the target lies at a sensor boundary.

➀ Data normalization

To streamline subsequent calculations, all datasets in Table 2 are converted into large-value indicator data. During the normalization process, the data are categorized into four types: large indicators (where higher values are preferable), small indicators (where lower values are better), intermediate indicators (optimal when values are close to a specific target), and interval indicators (best when values lie within a defined range).

**Figure 4:**
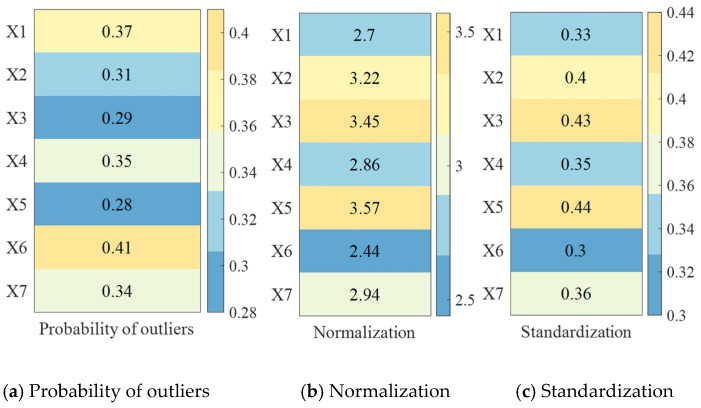
Heatmaps of results of normalization and standardization of probability of outliers.

The anomaly probability values shown in Figure 4a are normalized using Equation (6), resulting in an increasing metric that transforms into a very large indicator, as illustrated in Figure 4b.

The number of sensors covered is an interval metric, and the optimal interval is defined as [a,b], with a = 1 and b = 2. When normalizing the number of sensors covered in Figure 4a using Equation (6), in this paper, we take $Num=\max\{a-\min\{x_{i}\},\max\{x_{i}\}-b\}$ to obtain the values in Figure 4.

(6) $${\hat{x}}_{i}=\left\{\begin{array}{c} 1-\frac{a-x_{i}}{Num},x_{i}<a\\ 1,a\leq x_{i}\leq b\\ 1-\frac{x_{i}-b}{Num},x_{i}>b \end{array}\right.$$

(7) $$\varphi SOS(x_{i}{)}^{\prime }=\frac{1}{\varphi SOS(x_{i})}$$

➁ Data standardization

**Figure 5:**
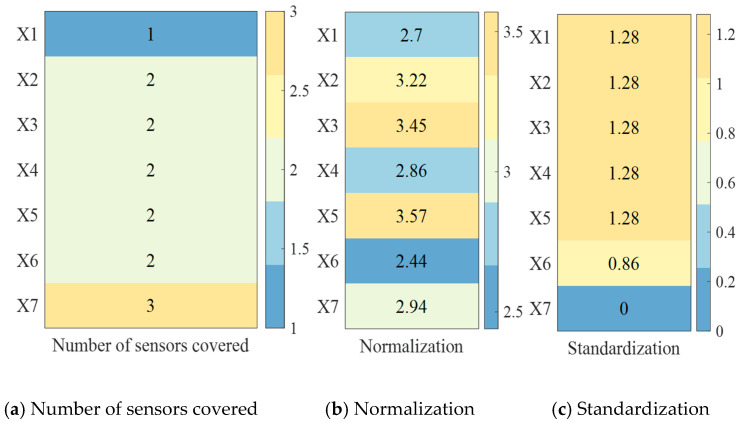
Heatmaps of results of normalization and standardization processes for number of sensors covered.

In order to eliminate the influence of different indicator dimensions, we standardize the forwarded matrix through Equation (8) to obtain the standardized outlier probability and number of sensors covered, as shown in Figure 5c.

(8) $$\varphi SOS(x_{i}{)}^{\prime }=\frac{1}{\varphi SOS(x_{i})}$$

where zi = [z_i1_,….z_im_] and vector zi denotes the ith target, *n* is the total number of targets to be detected, and m represents the evaluation metrics (outlier probability, number of sensors). The *n* target vectors form matrix (9), i.e., normalization matrix Z.

(9) $$Z=\left[\begin{array}{cc} z_{11} & z_{12}\\ z_{21} & z_{22}\\ : & :\\ z_{n1} & z_{n2} \end{array}\right]$$

➂ Calculation of optimal solution and worst solution

**Figure 6:**
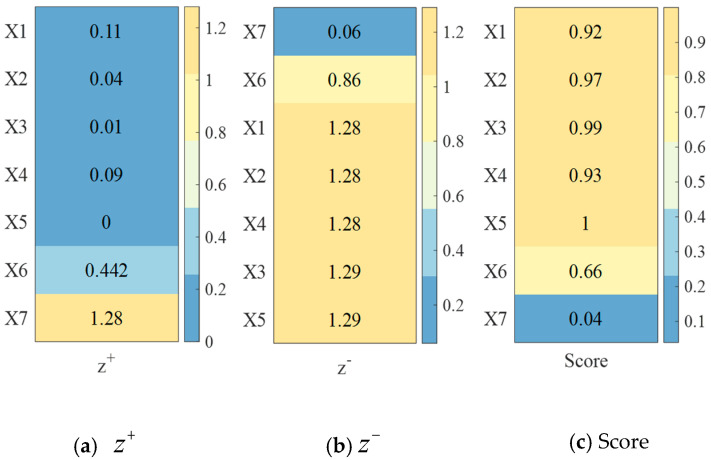
Heatmaps of optimal solution, worst solution, and approximation calculation results.

The ideal optimal solution vector is constructed by selecting the maximum value from each column of matrix (7). The distance from the ideal optimal solution of the ith objective zi is then computed using Equation (10). As is illustrated in Figure 6a, this process yields the optimal solution values corresponding to the seven targets observed by sensor 1.

(10) $$\begin{array}{l} z^{+}=\left[z_{1}^{+},z_{2}^{+}\right]=\left[\max\{z_{11},z_{21},\ldots ,z_{n1}\},\max\{z_{12},z_{22},\ldots ,z_{n2}\}\right]\\ \Rightarrow d_{i}^{+}=\sqrt{\sum_{j=1}^{2}{\left(z_{j}^{+}-z_{ij}\right)}^{2}} \end{array}$$

The smallest number in each column from matrix (10) is taken to form the ideal worst solution vector, and for the ith target zi, the distance between it and the worst solution is calculated using Equation (11). Figure 6b illustrates the worst solutions for the seven targets of sensor 1.

(11) $$\begin{array}{l} z^{-}=\left[z_{1}^{-},z_{2}^{-}\right]=\left[\min\{z_{11},z_{21},\ldots ,z_{n1}\},\min\{z_{12},z_{22},\ldots ,z_{n2}\}\right]\\ \Rightarrow d_{i}^{-}=\sqrt{\sum_{j=1}^{2}{\left(z_{j}^{-}-z_{ij}\right)}^{2}} \end{array}$$

➃ Calculation of relative proximity

Score Si for the ith target is calculated using Equation (12):

(12) $$S_{i}=\frac{d_{i}^{-}}{d_{i}^{+}+d_{i}^{-}}$$

Figure 6c shows a heatmap of score S_i_ obtained through Equation (12). In this paper, we sort S_i_ values from low to high. The higher the ranking, the more likely it is to be an outlier. Taking sensor 1 as an example, we set the number of targets to *n* and the number of outliers to *n* × 30%. Then, the abnormal targets for sensor 1 should be X6 and X7. The method proposed in this paper fully considers the situation when multiple sensors detect a target, especially when the target is located at the sensor detection boundary. By reasonably dividing the attribution of targets, repeated calculations are avoided and the accurate detection and identification of abnormal targets are ensured.

Conventional outlier handling often relies on fixed or manually tuned thresholds, which are suboptimal in environments with dynamic clutter. The proposed stochastic outlier selection (SOS) method adapts its rejection boundary based on local observation statistics and component confidence. This ensures better discrimination between true target-originated measurements and clutter in varying noise conditions.

3. TOPSIS-SOS clustering of GCs

Assuming that $G_{p}^{i},G_{q}^{j}$ are Gaussian components (GCs) representing different movement trajectories, we can compute their corresponding ranking scores based on the TOPSIS-SOS method. If the ranking position of a given GC $S(G_{p,q}^{i,j})$ is not within the top 30%, this implies that this component is not anomalous and is thus considered to belong to a shared cluster with others of similar scores. Accordingly, based on the clustering outcome of the TOPSIS-SOS analysis, the local intensity functions of nodes *I* and *j* can be represented as follows:

(13) $${\hat{D}}_{g}^{i,j}(x)=\sum_{g=1}^{G}{\hat{D}}_{g}^{i,j}(x)$$

When examining the Gaussian components (GCs) of nodes I and j within a given cluster, if the quantity $M_{g}^{i,j}$> 0, this indicates that there are shared GCs present in the cluster, and it thus becomes necessary to perform categorization or grouping of these GCs.

At this stage, the corresponding sub-intensity functions for the shared cluster can be expressed as follows:

(14) $${\hat{D}}_{g}^{i,j}(x)=\sum_{p=1}^{M_{g}^{i,j}}\alpha_{g,p}^{i}N(x;m_{g,p}^{i},P_{g,p}^{i})$$

If $M_{g}^{i,j,k}=0$, this implies that the cluster contains no Gaussian components (GCs), making classification unnecessary. In such cases, the related sub-intensity is directly represented by ${\hat{D}}_{g}^{i,j}(x)=0$. Based on the IID cluster density decomposition principle, the sub-intensity derived through the TOPSIS-SOS clustering procedure replaces that from the original state domain, denoted as $N_{k,g}=\left\{i:M_{g}^{i,j}>0\right\}\to N_{k,g}$. This ensures that only sensor nodes associated with a specific cluster contribute to its fusion process.

The overall procedure of applying the TOPSIS-SOS method to GC clustering can be outlined as follows: to integrate this clustering approach with multi-perspective boundary segmentation, it is necessary to determine the estimated target count ${M^{\prime }}_{g}^{i,j}(x)$ for each region.

(15) $${M^{\prime }}_{g}^{i,j}(x)=\sum_{o}\int_{x^{j}}{\hat{D}}_{g}^{i}(x)dx$$

The corresponding spatial target density is as follows:

(16) $$s_{g}^{i,j}(x)=\frac{{\hat{D}}_{g}^{i}(x)}{{M^{\prime }}_{g}^{i,j}(x)}$$

To calculate the segmentation base distribution, the initial step is to determine ${\hat{D^{\prime }}}_{g}^{i,j}(x)$. Utilizing the aforementioned clustering analysis in combination with boundary segmentation derived from varying sensor perspectives, the sub-intensity at this specific location can be formulated as follows:

(17) $${\hat{D^{\prime }}}_{g}^{i,j}(x,o)=\sum_{p:(i,p)\in c_{g}}{\hat{D}}_{g}^{i,j}(x,o)=\sum_{p:(i,p)\in c_{g}}\alpha_{g,p}^{i}G(x,m_{g,p}^{i},P_{g,p}^{i})$$

The partitioned region needs to be partitioned into $F^{j}$ and C together to generate $C^{\prime }$.

(18) $$\left({\left\{C_{g}\right\}}_{g=1}^{G}{)}^{\prime }\notin F_{s}^{T}(F_{s}^{T}=\underset{M=1:N}{\cup }F^{L_{M}}\right)$$

(19) $$C^{\prime }=({\left\{C_{g}\right\}}_{g=1}^{G}{)}^{\prime }$$

$C^{\prime }$ corresponds to the target RFS approximated as multi-Bernoulli with probability distribution $G_{p}^{i}(x)$, and there exists probability $\alpha_{p}^{i}\in [0,1]$. The formula for the basis distribution of sensor i in $F^{j}$ is ${\hat{D^{\prime }}}_{g}^{i,j}(x)$.

(20) $${p^{\prime }}_{g}^{i,j}(n)={\hat{p}}_{g}^{i,j}(n)=\prod_{p=1}^{M^{i,j}}\left(1-\alpha_{g,p}^{i,j}\right)\sigma_{M^{i,j},n}(\frac{\alpha_{g,1}^{i,j}}{1-\alpha_{g,1}^{i,j}},\cdots ,\frac{\alpha_{g,M^{i,j}}^{i,j}}{1-\alpha_{g,M^{i,j}}^{i,j}})$$

At this stage, $\sigma_{M^{i,j},n}(\cdot )$ represents a primitively symmetric function with respect to $M^{i,j},n$ repetitions.

Based on the properties inherent to the multi-Bernoulli multi-object probability density (MPD), the following conclusion can be drawn:

(21) $$\sum_{n=0}^{\infty }np_{i}^{j}(n)=\sum_{m=1}^{M_{i,j}}\alpha_{p}^{i}={M^{\prime }}_{g}^{i,j}$$

The original distribution can be reconstructed by convolving the cardinality distributions from each separate region:

(22) $$\sum_{n_{1}+n_{2}+\cdots n_{N}=n}\left[p_{i}^{1}(n_{1})\cdots p_{i}^{N}(n_{N})]=p_{i}(n\right)$$

#### 3.2.2. Fusion

In this study, for each partitioned region, the GM-CPHD base distributions $p_{i}^{j}(n)$ and ${\hat{D}}_{g}^{i,j}(x)$, obtained from different sensors, are individually fused using the GA (genetic algorithm) fusion strategy. The multi-target probability distribution (MPD) is represented as $f(X)=n!p(n)\prod_{m\in X}q(m)$, where q(m) denotes the single-target probability density, and p(n) captures the distribution over the number of targets.

Assume the UAV swarm operates within a network $f(X)=n!p(n)\prod_{m\in X}q(m)$, composed of N sensors. Let q(m) be the set of sensor nodes, and p(n) represent the communication links between them. For any pair of nodes i and j, if a communication link (i,j)∈A exists between them, and node j can receive data from node i, then node j is considered an internal node of i, denoted by $N^{i}=\left\{j\in N:(j,i)\in A\right\}$.

(23) $$f_{GA}(X)=\frac{\prod_{i\in N^{j}}{\left[f_{i}(X)\right]}^{\omega^{i}}}{\int \prod_{i\in N^{j}}{\left[f_{i}(X)\right]}^{\omega^{i}}\delta X}$$

Under the KLD variation principle, it becomes

(24) $$f_{GA}(X)=\underset{g(X):\int g(x)\delta X=1}{\arg\min}\sum_{i\in N^{j}}\omega_{i}D_{KL}(\left.g(X)\right\|f_{i}(X))$$

At each time step k, every sensor node carries out a predefined number of fusion (or concordance) iterations, denoted as L ≥ 1, to combine its local density with those from neighboring nodes. Under the assumption of GA fusion, the fusion operation for node $l+1^{th}$ at each step is expressed as follows:

(25) $$s_{g}^{i.l+1}(X)=\frac{\prod_{j\in Ni}{\left[s_{g}^{i.l+1}(X)\right]}^{\omega^{i,j}}}{\int \prod_{j\in N^{i}}{\left[s_{g}^{i.l+1}(X)\right]}^{\omega^{i,j}}dX}$$

(26) $$p_{g}^{i,l+1}(n)=\frac{\prod_{j\in N^{i}}{\left[p_{g}^{i,l}(n)\right]}^{\omega^{i,j}}{\left(\int \prod_{j\in N^{i}}{\left[s_{g}^{j,l}(X)\right]}^{\omega^{i,j}}dX\right)}^{n}}{\sum_{m=0}^{\infty }\prod_{j\in N^{i}}{\left[p_{g}^{j,l}(m)\right]}^{\omega^{i,j}}{\left(\int \prod_{j\in N^{i}}{\left[s_{g}^{j,l}(X)\right]}^{\omega^{i,j}}dX\right)}^{m}}$$

Within each region, the base distributions $p_{i}^{j}(n)$ $D_{i}^{j}(x,o)$ provided by the individual sensors are calculated independently, after which the GA fusion strategy is employed on the CPHD filter as follows:

(27) $$D_{GA}(x,o)=s_{GA}(x,o)\sum_{n=0}^{\infty }np_{GA}(n)$$

Among them,

(28) $$s_{GA}(x,o)=\frac{\sum_{m=0}^{\infty }\prod_{i\in N^{j}}{\left[s_{i}(x,o)\right]}^{w_{i}}}{\sum_{o}\int_{X}\prod_{i\in N^{j}}{\left[s_{i}(x,o)\right]}^{w_{i}}dx}$$

(29) $$p_{GA}(n)=\frac{\prod_{i\in N^{j}}{\left[p_{i}(n)\right]}^{w_{i}}{\left[\sum_{o}\int_{X}\prod_{i\in N^{j}}{\left[s_{i}(x,o)\right]}^{w_{i}}dx\right]}^{n}}{\sum_{m=0}^{\infty }\prod_{i\in N^{j}}{\left[p_{i}(m)\right]}^{w_{i}}{\left[\sum_{o}\int_{X}\prod_{i\in N^{j}}{\left[s_{i}(x,o)\right]}^{w_{i}}dx\right]}^{m}}$$

#### 3.2.3. Merging

To derive the multi-target probability density distribution of Gaussian components (GCs) across multiple sensors with differing viewpoints, the previously segmented multi-target densities from each distinct region need to be integrated. This integration is achieved by consolidating all fused sub-IID clustering results into one unified independent and identically distributed (IID) clustering process. By aggregating all sub-intensity functions and convolving their associated base distributions, the combined base distributions ${\bar{p}}_{i}^{j}(n)$ and ${\bar{D}}_{g}^{i,j}(x)$ for the GM-JMNS-CPHD filter are obtained as follows and the algorithm flowchart is shown in Algorithm 1:

(30) $${\bar{D}}_{g}^{i,j}(x)=\sum_{g=1}^{G}{\bar{D^{\prime }}}_{g}^{i,j}(x)$$

(31) $${\bar{p}}_{i}^{j}(n){=(\bar{p}}_{1}*{\bar{p}}_{2}\cdots {\bar{p}}_{G})(n)=\sum_{n_{1}+n_{2}+\cdots +n_{G}=n}{\bar{p}}_{1}(n_{1}){\bar{p}}_{2}(n_{2})\cdots {\bar{p}}_{G}(n_{G})$$

**Algorithm 1.** Multi-sensor, multi-perspective T-S-GM-JMNS-CPHD fusion algorithmInput:${\left\{G_{1,k}^{p}\right\}}_{p=1}^{M_{g}^{i}},{\left\{G_{2,k}^{p}\right\}}_{p=1}^{M_{g}^{j}}$, *β*,
$C_{1}={\left\{\left(1,p\right)\right\}}_{p=1}^{M_{g}^{i}}$,
$C_{2}={\left\{\left(1,p\right)\right\}}_{p=1}^{M_{g}^{i}}$,
$\;p_{i}^{j}(n),\;F_{i}$Each sensor i operates as a GM-JMNS-CPHD filterExecute T number of flooding communication iterations.for i∈N do for j=1:N do  for $M_{g}^{i,j,k}=1:g$
do   Find the particles positioned in the area of $F^{j}$   For $(i^{\prime },p^{\prime })\in C$ and $\left(i^{\prime },p^{\prime })\neq (i,p\right)$ do    Calculate $C={\left\{C_{g}\right\}}_{g=1}^{G}$ by Algorithm 1    Calculate $C^{\prime }=({\left\{C_{g}\right\}}_{g=1}^{G}{)}^{\prime },({\left\{C_{g}\right\}}_{g=1}^{G}{)}^{\prime }\notin F_{s}^{T}(F_{s}^{T}=\underset{M=1:N}{\cup }F^{L_{M}})N_{k,g}=\left\{i:M_{g}^{i,j}>0\right\}\to N_{k,g}$   end for  end for  Calculate ${{\hat{v}}^{\prime }}_{g}^{i,j}(x)$ by (17)  Calculate ${p^{\prime }}_{g}^{i,j}(n)$ by (20) end forend for  Calculate GA/AA fusion strategy
Calculate cardinality distribution ${\bar{p}}_{i}^{j}(n)$ and fusion target state density ${\bar{D}}_{g}^{i,j}(x)$ after merging by (30) and (31)Output: ${\bar{p}}_{i}^{j}(n){\bar{D}}_{g}^{i,j}(x)$

Traditional CPHD-based methods rely on a global Poisson assumption for cardinality, which may not accurately reflect spatially non-uniform target distributions. Our method decomposes the intensity into sub-regions and estimates cardinality using multi-Bernoulli modeling within each. This structure enables the filter to better model dense or sparse areas independently, thereby improving multi-target discrimination and reducing overestimation or underestimation.

#### 3.2.4. Algorithm Complexity Analysis

In general, the computation of the base distribution is independent of the specific model used [31,32,33,34]. This is because increasing the number of targets within the scene does not significantly impact the computational load for the base distribution. However, the computational complexity is relevant when considering CPHD and SOS-GM-JMNS-CPHD algorithms. The CPHD algorithm requires approximately m + 1 evaluations of the primitive logarithmic product function, leading to a complexity of $O(m^{3})∼O{\left|Z\right|}^{3}n_{\max})$, where m represents the number of evaluations and $n_{\max}$ is the maximum target count. When $n_{\max}>m$, CPHD can be regarded as $\left(m^{3}+n_{\max}m^{2}-\frac{m^{2}}{2})∼O(n_{\max}m^{2}\right)$. As was previously noted, the JMNS-CPHD’s computational complexity grows linearly with the number of modes, reaching $O({\left|Z\right|}^{3}n_{\max}O)$, where $n_{\max}$ denotes the maximum number of targets.

The T-S-GM-JMNS-CPHD algorithm does not change the base distribution computation of CPHD itself but influences the maximum target count through the clustering algorithm $n_{\max}\to {\bar{n}}_{\max}$. Although the clustering process adds complexity to the algorithm, it also modifies the maximum number of targets considered. The overall complexity of the T-S-GM-JMNS-CPHD algorithm can be expressed as $O({\left|Z\right|}^{3}n_{\max}O+O)∼O({\left|Z\right|}^{3}n_{\max}O)$, where the complexity increase is not due to issues like linear superposition as target numbers grow. Comparing the three methods—CPHD, GM-JMNS-CPHD, and T-S-GM-JMNS-CPHD the computational complexities are summarized as $O_{CPHD}<O_{GM-JMNS-CPHD}=O_{T-S-GM-JMNS-CPHD}$.

## 4. Simulation Result

To verify the effectiveness of the proposed T-S-GM-JMNS-CPHD algorithm, a GM-CPHD filter is employed to evaluate its tracking performance. The testing uses a nonlinear coordinated turn (CT) model, where the target is assumed to move on a two-dimensional plane at a constant turning rate. The system matrix describing this motion can be represented as follows:

(32) X(k+1)=F(k)X(k)+Γ(k)w(k)

Among them,

(33) $$F=\left[\begin{array}{cccc} 1 & 0 & \frac{\sin\omega T}{\omega } & -(\frac{1-\cos\omega T}{\omega })\\ 0 & 1 & \frac{1-\cos\omega T}{\omega } & \frac{\sin\omega T}{\omega }\\ 0 & 0 & \cos\omega T & -\sin\omega T\\ 0 & 0 & \sin\omega T & \cos\omega T \end{array}\right]$$

(34) $$\Gamma (k)=\left[\begin{array}{cc} 0.5T^{2} & 0\\ 0 & 0.5T^{2}\\ T & 0\\ 0 & T \end{array}\right],w(k)=\left[\begin{array}{c} w_{x}\\ w_{y} \end{array}\right]$$

The GM-JMNS-CPHD filter was configured with a sensor detection probability of 0.9 and a survival probability of 0.99. The average Poisson rate for uniform clutter affecting the moving target was set at 5, while the birth density was positioned at coordinates (±800 m, ±800 m). All simulations were conducted using 200 Monte Carlo runs. Within the GM-JMNS-CPHD filter, the truncation threshold was set to $T=10^{-5}$, the merging threshold to U=2, and the maximum number of allowable Gaussian components to $J_{\max}=100$. To evaluate the tracking performance for moving targets, the optimal sub-pattern assignment (OSPA) distance metric was used, with a parameter cutoff of c = 100 and order of *p* = 1. The parameters are shown in Table 3.

### 4.1. Comparison of Algorithms Applied to Simulation in Multiple Scenarios

To evaluate the performance of the proposed algorithm, we conducted simulation experiments across multiple tracking scenarios. The T-S-GM-JMNS-CPHD algorithm was implemented using a genetic algorithm (GA)-based fusion strategy to enhance its multi-target tracking capabilities. To assess the algorithm’s practicality and adaptability, several multi-sensor, multi-target tracking configurations were designed. Specifically, we examined three experimental setups: 2 sensors with 6 motion trajectories, 2 sensors with 15 motion trajectories, and 16 sensors with 15 motion trajectories. These varying scenarios allowed for a comprehensive analysis of the algorithm’s effectiveness under different levels of sensor complexity and target density.

**Figure 7:**
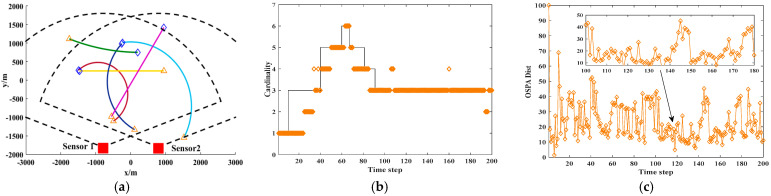
Experimental algorithm results in case of 2 sensors and 6 motion trajectories. (**a**) Real track of moving target. (**b**) Cardinality estimation result of moving track. (**c**) OSPA estimation result of moving track.

**Figure 8:**
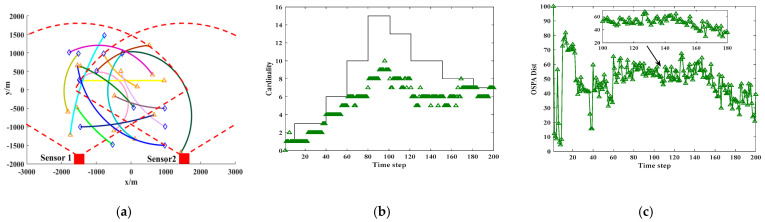
Experimental algorithm results in case of 2 sensors and 15 motion trajectories. (**a**) Real track of moving target. (**b**) Cardinality estimation result of moving track. (**c**) OSPA estimation result of moving track.

**Figure 9:**
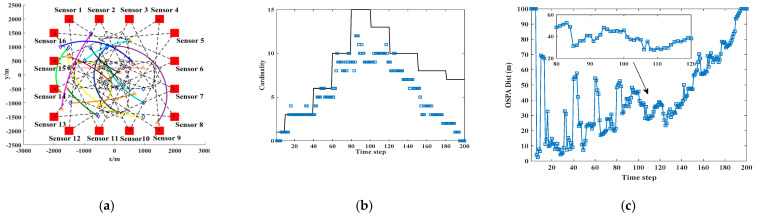
Experimental algorithm results in case of 16 sensors and 15 motion trajectories. (**a**) Real track of moving target. (**b**) Cardinality estimation result of moving track. (**c**) OSPA estimation result of moving track.

The experiments were Independently conducted to evaluate the applicability of the algorithm in various multi-sensor and multi-target nonlinear tracking scenarios. As is illustrated in Figure 7, Figure 8 and Figure 9, the T-S-GM-JMNS-CPHD algorithm demonstrated effective performance in handling both multi-sensor tracking and the tracking of multiple targets exhibiting nonlinear motion.

Furthermore, to assess the algorithm’s robustness across diverse environments, we examined its performance under varying object detection probabilities and different levels of uniform clutter, represented by the average Poisson value $\lambda_{c}$. By adjusting parameters $\lambda_{c}=5,8,10,20$ and $p_{D}=0.50,0.65,0.85,0.95$, we tested how well the algorithm adapts to multi-scene nonlinear tracking tasks. The results confirm that the proposed method maintains a strong tracking accuracy and robustness across different sensing conditions.

**Figure 10:**
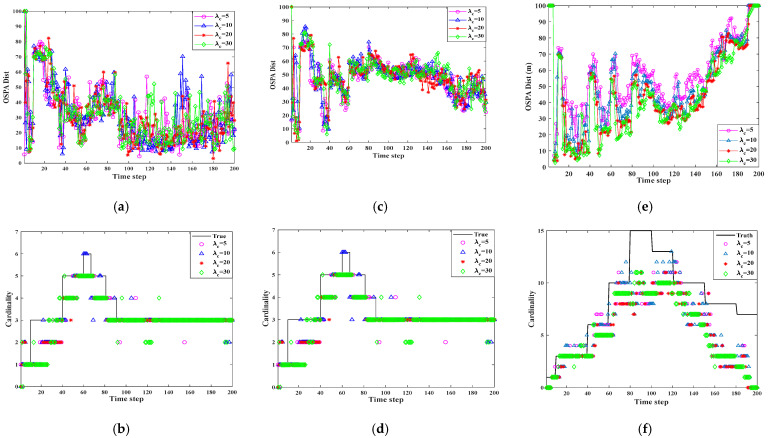
Comparison results of different scenarios T-S-GM-MJNS-CPHD with different $\lambda_{c}$. (**a**) Scenario 1: OSPA metric over time. (**b**) Scenario 1: cardinality estimates over time. (**c**) Scenario 2: OSPA metric over time. (**d**) Scenario 2: cardinality estimates over time. (**e**) Scenario 3: OSPA metric over time. (**f**) Scenario 3: cardinality estimates over time.

**Figure 11:**
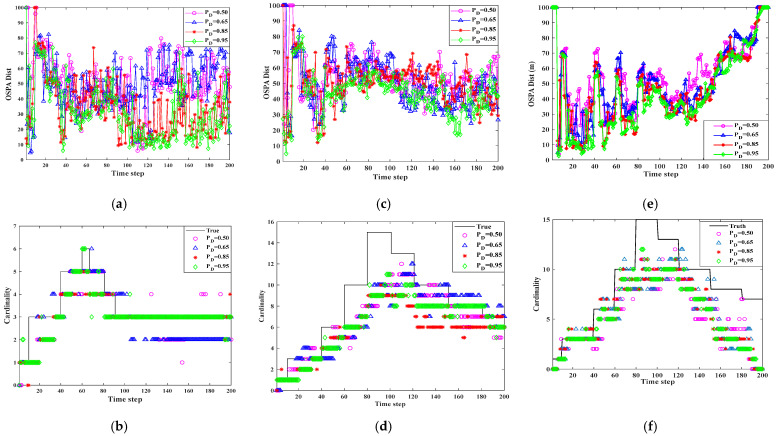
Comparison results of T-S-GM-MJNS-CPHD applied to different scenarios with different $p_{D}$. (**a**) Scenario 1: OSPA metric over time. (**b**) Scenario 1: cardinality estimates over time. (**c**) Scenario 2: OSPA metric over time. (**d**) Scenario 2: cardinality estimates over time. (**e**) Scenario 3: OSPA metric over time. (**f**) Scenario 3: cardinality estimates over time.

Figure 10 and Figure 11 show the results of verifying the performance of the algorithm at time $p_{D}=0.95$ and $\lambda_{c}=5,8,10,20$, in the three scenarios of 6 motion trajectories with 2 sensors, 15 motion trajectories with 2 sensors, and 15 motion trajectories with 16 sensors. The results show the following:

- In the three scenarios, the OSPA of multi-sensor multi-target tracking receives different object detection probabilities $\lambda_{c}$.
- There is a significant difference between the OSPA values produced for $\lambda_{c}=5$ and $\lambda_{c}=20$.

The T-S-GM-JMNS-CPHD algorithm will be affected by $\lambda_{c}$ during the process, and the effect will not change significantly with increased numbers of motion trajectories of the detected motion targets and sensors.

**Figure 12:**
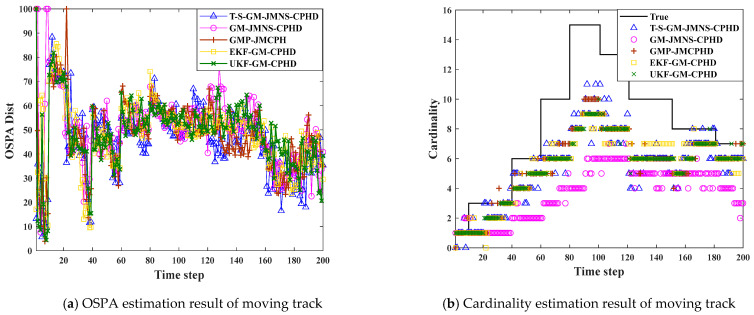
Comparison results between T-S-GM-MJNS-CPHD and other algorithms.

Figure 11 and Figure 12 show the results of verifying the performance of the SOS-GM-JMNS-CPHD algorithm at time $\lambda_{c}=10$ and $p_{D}=0.50,0.65,0.85,0.95$, with the SOS-GM-JMNS algorithm in three scenarios: 2 sensors with 6 motion trajectories, 2 sensors with 16 motion trajectories, and 16 sensors with 1 motion trajectories. The results show the following:

- In the three scenarios, the OSPA of multi-sensor multi-target tracking will be more affected by different object detection probabilities, $p_{D}$.
- There is a significant difference between the OSPA values produced for $p_{D}=0.50$ and $p_{D}=0.95$.
- The T-S-GM-JMNS-CPHD algorithm is strongly influenced by $p_{D}=0.95$, and that influence does not change significantly with increased numbers of motion trajectories of the detected motion targets and sensors.

Comparing the different scenarios mentioned above, it can be seen that there is a significant difference in OSPA values between $\lambda_{c}=5$ and $\lambda_{c}=20$. There is a significant difference in OSPA values between $p_{D}=0.50$ and $p_{D}=0.95$. The SOS-GM-CPHD algorithm is somewhat influenced by $\lambda_{c}$ $p_{D}$ during use, and this influence does not significantly change with the increase in detected moving-target trajectories or the number of sensors.

### 4.2. Comparison of the Algorithm with Other Algorithm Simulations

To evaluate the effectiveness of the proposed algorithm, we conducted comparative experiments using multiple filtering methods under various scenarios. The performance of each algorithm was assessed based on key metrics such as estimation accuracy, robustness to noise and clutter, and computational efficiency. GM-JMNS-CPHD filtering can be applied to nonlinear systems, but the effect of CPHD directly acting on nonlinear systems is not very good. The SOS algorithm artificially sets the threshold, and the impact of the TOPSIS-SOS directly acting on the GM-JMNS-CPHD system is determined by whether the threshold selection is correct. Therefore, this study selects GM-JMNS-CPHD filtering as the measure for research. By implementing these filters under identical conditions, we were able to perform a fair and comprehensive comparison, highlighting the advantages and limitations of each approach. We compared the effect of T-S-GM-JMNS-CPHD, GM-JMNS-CPHD, and GMP-JMCPHD in [30], and extended Kalman filter (EKF)-GM-CPHD and unscented Kalman filter (UKF)-GM-CPHD in [35]. We set $p_{D}=0.95$ and $\lambda_{c}=10$ for two sensors with 15 motion trajectories to carry out the analysis. As the comparison results in Figure 12 show, the differences between the proposed T-S-GM-JMNS-CPHD algorithm and several other algorithms may be mainly due to the effects of the other algorithms, the results of the study approximating the GMP-MJCPHD, and the comparative effectiveness of the other methods.

Under non-overlapping FoV conditions, traditional fusion algorithms may suffer from redundant or incomplete information due to misalignment of the sensor coverage. By designing a state-space-partitioned fusion structure, the proposed framework assigns local estimation responsibility to different sensors based on the spatial domain division. This not only reduces fusion conflict but also ensures that global target states are reconstructed more efficiently and consistently, especially when targets move across sensor boundaries.

## 5. Conclusions

In this paper, we propose a nonlinear multi-target tracking method based on Gaussian mixture–jump Markov–CPHD fusion with non-identical fields of view. Firstly, we explored the effects of sensors with different FoVs on the fusion of nonlinear motion targets for multi-sensor tracking. Secondly, we used the SOS ordering method to approximate the ideal solution, and simultaneously performed normalization, standardization, optimal-solution, and worst-solution computations, and calculated the relative proximity of the outlier data and the number of sensors covered by the different viewpoints, so as to improve the accuracy of outlier detection. The posterior intensity function was partitioned into several sub-intensity components, and the distribution of target counts in each region was modeled using a multi-Bernoulli reconstruction based on the underlying base distribution. Simulation outcomes demonstrate that the proposed T-S-GM-JMNS-CPHD algorithm exhibits a strong robustness and reliable performance in complex multi-target tracking environments, particularly where sensors have varying fields of view. When compared with existing approaches such as the GM-JMNS-CPHD, GMP-MJCPHD, EKF-GM-CPHD, and UKF-GM-CPHD algorithms, the T-S-GM-JMNS-CPHD method consistently delivers superior effectiveness in handling nonlinear motion tracking tasks.

The proposed method assumes a static sensor with a uniform field of view and does not consider changes in sensor position or orientation over time. Additionally, the algorithm has not yet been tested in scenarios involving heterogeneous sensor networks or mobile platforms. These assumptions may limit its applicability in real-world systems where dynamic sensor configurations are common. In future work, we plan to extend the current framework to handle time-varying FoVs, multi-sensor fusion, and adaptive modeling techniques to support heterogeneous sensing environments. Integration with sensor planning and control strategies will also be explored to improve real-time performance.

## Figures and Tables

**Table 1:** Initial state of target in nonlinear Gaussian measurement model.

Target	Initial State	Appearing Frame	Disappearing Frame
1	[−250 − 5.8857. 20. 1000 + 11.4102. 3. − wturn/3]	1	truth.K + 1
2	[−1500 − 7.3806. 11. 250 + 6.7993. 10. − wturn/2]	10	truth.K + 1
3	[−1500. 43. 250. 0. 0]	10	66
4	[−250 + 7.3806. − 12. 1000 − 6.7993. − 12. wturn/3]	40	truth.K + 1
5	[250. − 50. 750. 0. − wturn/4]	40	80
6	[1000. − 50. 1500. − 80. 0]	60	90

**Table 2:** Initial target state for a nonlinear Gaussian measurement model.

Target	Outlier Probability	Number of Sensors Covered
X1	0.37	1 + 0.5
X2	0.31	2
X3	0.29	2
X4	0.35	2
X5	0.28	2
X6	0.41	2 + 0.5
X7	0.34	3 + 0.5

**Table 3:** Initial target state for a nonlinear Gaussian measurement model.

Category	Parameter/Description	Category	Parameter/Description
Programming Environment	MATLAB R2023a	Birth Density Coordinates	(±800 m, ±800 m)
Monte Carlo Trials	200 runs	Truncation Threshold	$T=10^{-5}$
Sensor Detection Probability	0.9	Merging Threshold	U=2
Survival Probability	0.99	Max Gaussian Components	$J_{\max}=100$
Clutter Rate (Poisson Avg.)	5	Evaluation Metric	OSPA (c = 100, *p* = 1)

## Data Availability

The original contributions presented in this study are included in the article. Further inquiries can be directed to the corresponding author.

## References

[1] Trinh M.L., Nguyen D.T., Dinh L.Q., Nguyen M.D., Setiadi D.R.I.M., Nguyen M.T. (2025). Unmanned Aerial Vehicles (UAV) Networking Algorithms: Communication, Control, and AI-Based Approaches. Algorithms.

[2] Lei X., Hu X., Wang G., Luo H. (2023). A Multi-UAV Deployment Method for Border Patrolling Based on Stackelberg Game. J. Syst. Eng. Electron..

[3] Gargalakos M. (2024). The Role of Unmanned Aerial Vehicles in Military Communications: Application Scenarios, Current Trends, and Beyond. J. Def. Model. Simul..

[4] Dagan O., Cinquini T.L., Ahmed N.R. Non-Linear Heterogeneous Bayesian Decentralized Data Fusion. Proceedings of the 2023 IEEE/RSJ International Conference on Intelligent Robots and Systems (IROS).

[5] Vo B.T., Vo B.N., Cantoni A. (2007). Analytic Implementations of the Cardinalized Probability Hypothesis Density Filter. IEEE Trans. Signal Process..

[6] Bao F., Zhang Z., Zhang G. (2024). An ensemble score filter for tracking high-dimensional nonlinear dynamical systems. Comput. Methods Appl. Mech. Eng..

[7] Liu J., Cheng G., Song S. (2025). Event-triggered distributed diffusion robust nonlinear filter for sensor networks. Signal Process..

[8] Cheng C., Tourneret J.-Y., Yıldırım S. (2025). A variational Bayesian marginalized particle filter for jump Markov nonlinear systems with unknown measurement noise parameters. Signal Process..

[9] Wu Y., Qian W. (2025). Adaptive memory-event-triggered-based double asynchronous fuzzy control for nonlinear semi-Markov jump systems. Fuzzy Sets Syst..

[10] Shen H., Wang G., Xia J., Park J.H., Xie X.-P. (2025). Interval type-2 fuzzy H∞ filtering for nonlinear singularly perturbed jumping systems: A semi-Markov kernel method. Fuzzy Sets Syst..

[11] Takata H., Komatsu K., Narikiyo K. (2023). A Nonlinear Filter of EKF Type Using Formal Linearization Method. IEEJ Trans. Electr. Electron. Eng..

[12] Zhu J., Xie Z., Zhao Y.B., Dullerud G.E. (2023). Event-triggered asynchronous filtering for networked fuzzy non-homogeneous Markov jump systems with dynamic quantization. Int. J. Adapt. Control Signal Process..

[13] Oliveira A.M.D., Santos S.R.B., Costa O.L.V. (2023). Mixed Reduced-Order Filtering for Discrete-Time Markov Jump Linear Systems With Partial Information on the Jump Parameter. IEEE Trans. Syst. Man Cybern. Syst..

[14] Sun Y.C., Kim D., Hwang I. (2022). Multiple-model Gaussian mixture probability hypothesis density filter based on jump Markov system with state-dependent probabilities. IET Radar Sonar Navig..

[15] Tao J. (2023). Event-Triggered Control for Markov Jump Systems Subject to Mismatched Modes and Strict Dissipativity. IEEE Trans. Cybern..

[16] Yu X., Feng X.A. (2024). Joint multi-Gaussian mixture model and its application to multi-model multi-bernoulli filter. Digit. Signal Process..

[17] Wang G.Q., Li N., Zhang Y.G. (2021). Distributed maximum correntropy linear and nonlinear filters for systems with non-Gaussian noises. Signal Process..

[18] Zhou Y., Zhao J., Wu S., Liu C. (2024). A Poisson multi-Bernoulli mixture filter for tracking multiple resolvable group targets. Digit. Signal Process..

[19] Lan L., Wei L.G. (2025). Zonotopic distributed fusion for 2-D nonlinear systems under binary encoding schemes: An outlier-resistant approach. Information Fusion.

[20] Hu Z., Guo T. (2025). Distributed resilient fusion filtering for multi-sensor nonlinear singular systems subject to colored measurement noises. J. Frankl. Inst..

[21] Zhao L., Sun L., Hu J. (2024). Distributed nonlinear fusion filtering for multi-sensor networked systems with random varying parameter matrix and missing measurements. Neurocomputing.

[22] Luo R., Hu J., Dong H.L., Lin N. (2025). Fusion filtering for nonlinear rectangular descriptor systems with Markovian random delays via dynamic event-triggered feedback. Commun. Nonlinear Sci. Numer. Simul..

[23] Jin Y.W., Lu X.Y., Li J. (2024). Multisensor multitarget distributed fusion for discrepant fields of view. Digit. Signal Process..

[24] Chen F., Nguyen H.V., Leong A.S. (2025). Sabita Panicker, Robin Baker, Ranasinghe, D.C. Distributed multi-object tracking under limited field of view heterogeneous sensors with density clustering. Signal Process..

[25] Li G.C., Battistelli G., Yi W., Kong L.J. (2020). Distributed multi-sensor multi-view fusion based on generalized covariance intersection. Signal Process..

[26] Wang L., Zhao J., Shi L., Zhang J. (2024). A GM-JMNS-CPHD Filter for Different-Fields-of-View Stochastic Outlier Selection for Nonlinear Motion Tracking. Sensors.

[27] Wang L., Chen G., Zhang L., Wang T. (2024). Stochastic Outlier Selection via GM-CPHD Fusion for Multitarget Tracking Using Sensors With Different Fields of View. IEEE Sens. J..

[28] Vo B.N., Pasha A., Tuan H.D. A Gaussian Mixture PHD Filter for Nonlinear Jump Markov Models. Proceedings of the 45th IEEE Conference on Decision and Control.

[29] Mahler R. On multitarget jump-Markov filters. Proceedings of the 2012 15th International Conference on Information Fusion.

[30] Da K., Li T., Zhu Y., Fu Q. (2020). Gaussian Mixture Particle Jump-Markov-CPHD Fusion for Multitarget Tracking Using Sensors With Limited Views. IEEE Trans. Signal Inf. Process. Over Netw..

[31] Kabiri S., Lotfollahzadeh T., Shayesteh M.G., Kalbkhani H., Solouk V. (2016). Technique for order of preference by similarity to ideal solution based predictive handoff for heterogeneous networks. IET Commun..

[32] He L., Mohammad Y., Cheng G.H., Peng W. (2022). A reliable probabilistic risk-based decision-making method: Bayesian Technique for Order of Preference by Similarity to Ideal Solution (B-TOPSIS). Soft Comput. A Fusion Found. Methodol. Appl..

[33] Gaeta A., Loia V., Orciuoli F. (2024). An explainable prediction method based on Fuzzy Rough Sets TOPSIS and hexagons of opposition: Applications to the analysis of Information Disorder. Inf. Sci..

[34] Fernández N., Bella J., Dorronsoro J.R. (2022). Supervised outlier detection for classification and regression. Neurocomputing.

[35] Mahler R. (2007). PHD filters of higher order in target number. IEEE Trans. Aerosp. Electron. Syst..

